# Two years of SARS-CoV-2 infection (2019–2021): structural biology, vaccination, and current global situation

**DOI:** 10.1186/s43162-021-00092-7

**Published:** 2022-01-14

**Authors:** Waqar Ahmad, Khadija Shabbiri

**Affiliations:** 1 Department of Biochemistry, College of Medicine and Health Sciences, UAE University, Al Ain, United Arab Emirates; 2 The University of Queensland, Brisbane, Australia

**Keywords:** SARS-CoV-2, COVID-19, Structural biology, Epidemiology, Vaccination, Drugs, Risk factors

## Abstract

The deadly SARS-CoV-2 virus has infected more than 259,502,031 confirmed cases with 5,183,003 deaths in 223 countries during the last 22 months (Dec 2019–Nov 2021), whereas approximately 7,702,859,718, vaccine doses have been administered (WHO: https://covid19.who.int/) as of the 24th of Nov 2021. Recent announcements of test trial completion of several new vaccines resulted in the launching of immunization for the common person around the globe highlighting a ray of hope to cope with this infection. Meanwhile, genetic variations in SARS-CoV-2 and third layer of infection spread in numerous countries emerged as a stronger prototype than the parental. New and parental SARS-CoV-2 strains appeared as a risk factor for other pre-existing diseases like cancer, diabetes, neurological disorders, kidney, liver, heart, and eye injury. This situation requires more attention and re-structuring of the currently developed vaccines and/or drugs against SARS-CoV-2 infection. Although a decline in COVID-19 infection has been reported globally, an increase in COVID-19 cases in the subcontinent and east Mediterranean area could be alarming. In this review, we have summarized the current information about the SARS-CoV-2 biology, its interaction and possible infection pathways within the host, epidemiology, risk factors, economic collapse, and possible vaccine and drug development.

## Introduction

In late 2019, several cases of humans infected with a novel coronavirus were first reported in China. This virus was named severe acute respiratory syndrome coronavirus 2 (SARS-CoV-2) and is commonly known as COVID-19 [1]. Soon after the emergence, this virus spread throughout the world within a short time period. The virus was able to be transferred from one person to another through respiratory droplets or by touch. Although the genetic structure of SARS-CoV-2 was very close to the other coronaviruses, the drugs proposed based on previous experiences were not much effective for these species. Meanwhile, the ultimate spread of this virus globally highlighted the urgent need for an effective vaccine against this pandemic until the remedy/ drug availability [2]. However, to develop an effective drug or vaccination against such a pathogen requires comprehensive details of the pathogen structure, its interaction with the host, and mode of action. Unlike a simple pathogen, the host, especially humans, has a complex cellular mechanism and clinical manifestations caused by SARS-CoV-2 were vibrant within different age groups and ethnic groups. Due to the complex nature of the human cellular system, it is possible that the presence of other diseases or body abnormalities might enhance the chance of incidence or severity of SARS-CoV-2 infection [2, 3]. In this study, we tried to summarize the published data on the biology of SARS-CoV-2 and its interaction with the host, possible drug and vaccine development, global economic situation, current epidemiology, and associated risk factors.

## SARS-CoV-2/ COVID-19 structural biology

### SARS-CoV2 genome

**Fig. 1:**
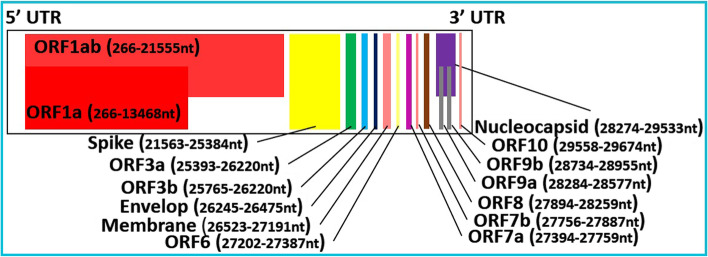
Genome structure of SARS-CoV-2. The genome of SARS-CoV2 comprises of 5′ and 3′ untranslated region (UTR) and several open reading frames (ORFs) comprising of non-structural and structural proteins including spike, envelop, membrane, and nucleocapsid

SARS-CoV-2 was declared as a “novel virus” due to not completely matched genome with previously found species causing similar symptoms based on PCR and next-generation techniques’ generated results. SARS-CoV-2 belongs to the *Betacoronovirus* family on the basis of conserved 1a and 1ab protein-encoding open reading frame (ORF) sequence. The SARS-CoV-2 reference sequence submitted to the GISAID (https://www.gisaid.org/epiflu-applications/hcov-19-reference-sequence/) database has been considered as the official reference sequence and named hCoV-19/Wuhan/WIV04/2019 **(WIV04)**. This reference sequence (total length: 29903nt) can also be downloaded from NCBI accession number NC_045512. This sequence showed almost an 80% similarity with previously known SARS-CoV [1] whereas a 96% similarity to a bat coronavirus at the whole-genome level [2]. Figure 1 is adopted from GISAID and depicted a current genome structure of SARS-CoV-2.

Briefly, ORF1ab is the longest ORF, approximately covering 2/3 of the whole genome. It has a position from 266 to 21,555 nt with a 21,289-nt length. It contains ORF1ab polyprotein of 7096AA and could be cleaved into several non-structural proteins ranging from NSP1-NSP16. The second-largest ORF1a is a part of ORF1ab and has a length of 13,202 nt from 266 to 13,468 nt. The ORF1a polyprotein could result in several NSPs (NSP1–NSP10) via proteolytic cleavage. ORF1ab is followed by spike glycoprotein (position 21,563 to 25,384, length: 3821nt), ORF3a (position 25,393 to 26,220, length: 827 nt, NS3a protein of 275AA), ORF3b (position 25,765 to 26,220, length: 455nt, NS3b protein of 151AA), Envelop protein (position 26,245 to 26,472, length: 227 nt, 75AA), membrane protein (position 26,523 to 27,191, length: 668 nt, 222AA), ORF6/NS6 (position 27,202 to 27,387, length: 185 nt, 61AA), ORF7a/ NS7a (position 27,394 to 27,759, length: 365 nt, 121AA), ORF7b/NS7b (position 27,756 to 27,887, length: 131 nt, 43AA), ORF8/NS8 (position 27,894 to 28,259, length: 365 nt, 121AA), nucleocapsid protein (position 28,274 to 229,533, length: 1259 nt, 419AA) with overlapped ORF9a/ NS9a (position 28,284 to 28,577, length: 239 nt, 97AA) and ORF9b/NS9b (position 28,734 to 28,955, length: 221 nt, 73AA), and at the end ORF10/ NS10 (position 29,558 to 29,674, length: 116 nt, 38AA).

### SARS-CoV-2 proteins and overall structure

**Fig. 2:**
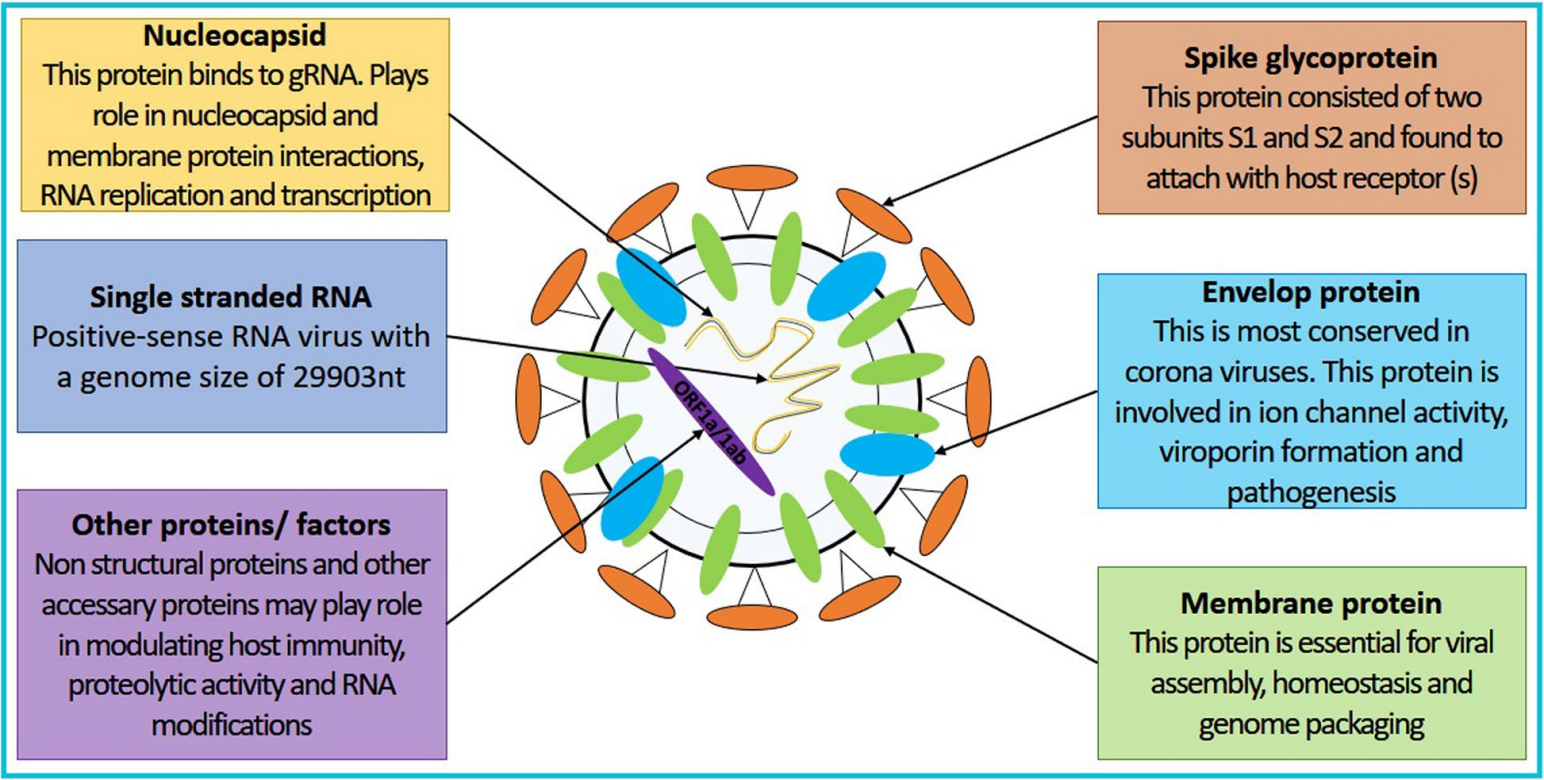
SARS-CoV-2 structure. SARS-CoV-2 is a positive-sense RNA virus consisting of four structural proteins namely envelop, membrane, nucleocapsid, and spike. It also has 16 non-structural proteins and 9 accessory proteins

The ORFs of SARS-CoV-2 inside the host are translated into 16 non-structural, 9 accessory, and 4 structural proteins (Fig. 2). For example, ORF1a and 1ab translated into polyproteins that further cleaved into 16 non-structural proteins (nps). These nps are essential for the virus life cycle into the host and may modulate host cellular immunity and proteolytic activity and may be involved in RNA synthesis, proofreading, and modification. In coronaviruses, the accessory proteins may play a role in modulating the host immune response such as regulation of inflammation markers, cell apoptosis, and ion channel activity. There are seven accessory protein-encoding ORFs in SARS-CoV-2, namely 3a, 6, 7a, 7b, 8, 9a, 9b, and 10. SARS-CoV-2 structural proteins including spike, envelop, membrane and nucleocapsid are necessary for viral structure maintenance and stability. A detailed explanation of these components can be extracted from a recent review by Kadam et al. [3].

### Virus- host interaction

**Fig. 3:**
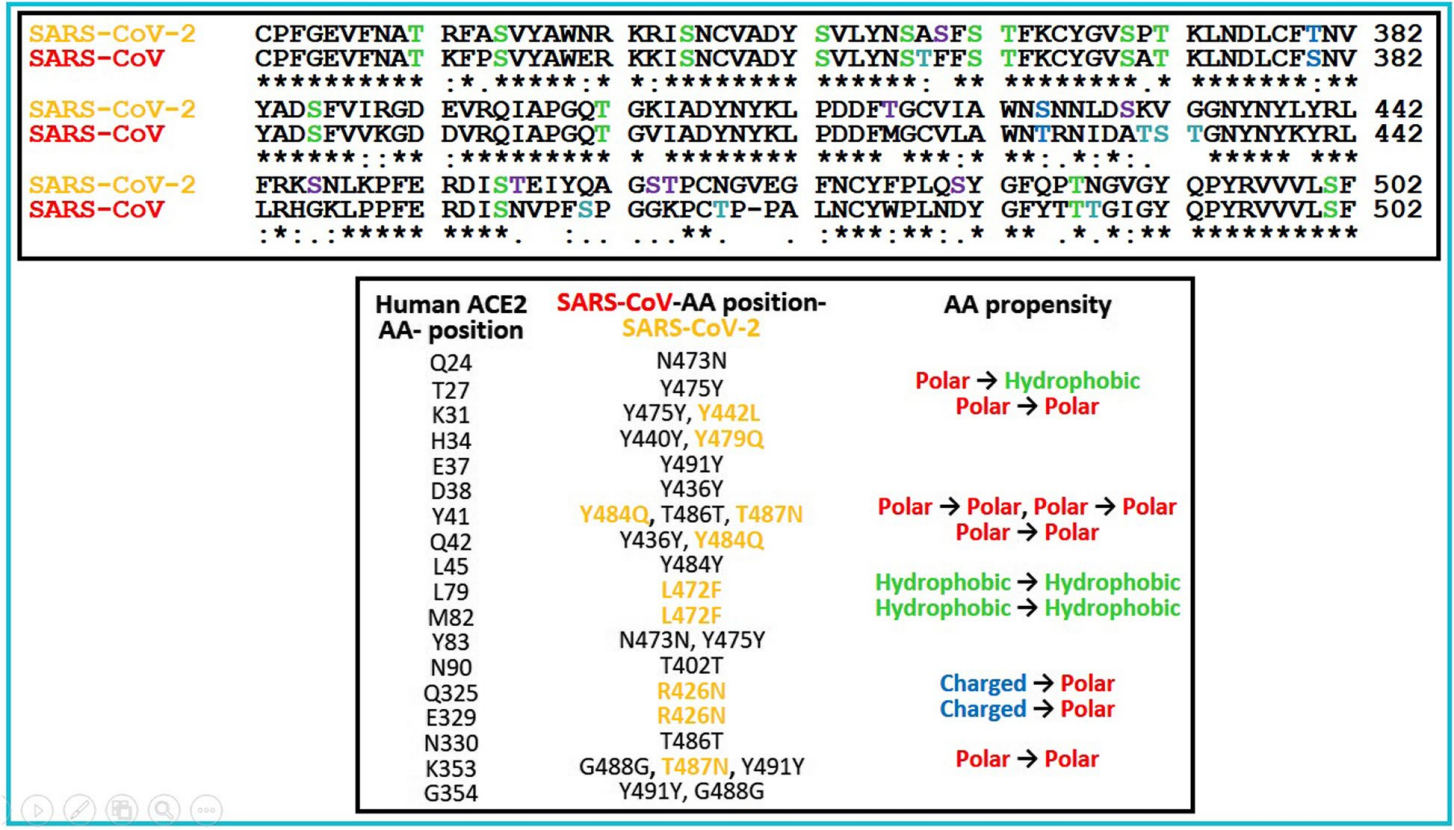
Sequence alignments and characterization of SARS-CoV-2 RBD. **a** Amino acid alignment for RBD of SARS-CoV-2 and SARS-CoV. Highlighted residues: green, conserved Ser/Thr between two sequences; blue, conserved substitution; pink, new Ser/ Thr residues introduced in SARS-CoV-2; sea green, Ser/Thr residues in SARS-CoV replaced by other amino acids in SARS-CoV-2. **b** Detailed binding interface of predicted SARS-CoV-2 RBD with ACE2 N-terminal. Possible changes in AAs are highlighted in yellow

Multiple studies had already discussed a detailed mechanism involved in the interaction of SARS-CoV-2 with host cells [2, 4–12]. Briefly, the first step in the viral infection is the host-receptor recognition. Previous studies [5–7, 13] showed a high affinity of SARS-CoV spike protein with human angiotensin-converting enzyme 2 (hACE2). The SARS transmembrane spike glycoprotein consists of two subunits S1 and S2. S1 is responsible for binding to the host receptor while S2 plays a role in the viral and cellular membrane fusions. It has been found that the receptor-binding domain of SARS-CoV-2 has a low affinity with hACE2 when compared to SARS-CoV. This could be due to the mutations and the structural difference between two RBDs. The SARS-CoV-2 spike sequence was aligned with the already published sequence of RBD from SARS-CoV (AA 323-502: PDB ID: 2AJF [14]) and this alignment resulted in a 72.2% identity and an 80% similarity with 1 gap (Fig. 3a). Sequence alignment showed a replacement of many previously determined critical residues [14] with others that may change SARS-CoV-2-ACE2 RBD structure-function insights. The following replacements in SARS-CoV-2 were found when compared to SARS-CoV: T424S, R425N, I427L, A429S, T430K, S431V, T432G, K438L, L443F, H445K, G446S, K447N, N457T, V458Em P459I, F460Y, S461Q, P462A, D463G, G464S, K465T, T468N, P469G, P470E, A471G, L472F, W476F, N479Q, D480S, Y484Q, T485P, T487N, and I489V (Fig. 3b). To further explore the role of these amino acid changes in SARS-CoV-2, a map was constructed representing any substantial differences between ACE2 and SARS-CoV-2 contact points [15]. This map was constructed using already published data and a total of 14 spike residues of the SARS-CoV showed interactions with 18 residues of the ACE2 receptor protein [14]. These changes may alter the hydrophobic association between SARS-CoV-2 and receptor ACE2. These results showed changes in the following contacts: Y442L mutation will change interaction from polar to hydrophobic with ACE2-Lys31, there was no change in polarity at ACE2 H34, Y41, Q42 and K353 contacted residues with SARS-CoV-2 after mutations at Y479Q, Y484Q, T487N, and L472F, respectively. The mutation at R426N will change interactions with ACE2 Q325 and E329. These changes may alter the interaction between ACE2 and SARS-CoV-2 and could be used as possible drug targets [15]. Although SARS-CoV-2 showed a lower affinity with hACE2 when compared to SARS-CoV, hACE2 is still a well-recognized host receptor for SARS-CoV-2. The other mechanism involved during host entry is host-protease activation. Shang et al. [13] found that furin pre-activation induced SARS-CoV-2 pseudovirus entry into different cell lines with ACE2 receptors. Moreover, SARS-CoV-2 pseudovirus entry was also activated through the cell-surface protease TMPRSS2 (transmembrane protease serine 2) and the lysosomal cathepsins. These observations make both hACE2 and TMPRSS2 possible drug targets to inhibit SARS-CoV-2 entry in human cells.

## Mode of transmission

Generally, there were three phases involved in the SARS-CoV-2 pandemic, including local outbreak, community transmission, and large-scale transmission [16]. SARS-CoV-2 has been considered as a respiratory virus and the infected persons have been found upper respiratory symptoms like sneezing and coughing. Below are given main transmission routes for the SARS-CoV-2 in humans according to WHO and other sources (https://www.who.int/news-room/commentaries/detail/transmission-of-sars-cov-2-implications-for-infection-prevention-precautions).

### Person to person contact and respiratory droplets

Contamination of the respiratory droplets via a person-to-person contact has been found as one of the major infection routes for the SARS-CoV-2. Respiratory droplets are of a 5–10-μm diameter in size and can be transmitted to another person while coughing, sneezing, talking, or singing [17].

### Airborne transmission

Airborne transmission is a spread of an infectious agent caused by the diffusion of aerosols containing infection in the air over a long period of time and distance. The sources or producers of the SARS-CoV-2 airborne transmission may include generation of aerosols during a medical procedure of an infected patient, indoor settings with poor ventilation, and inhalation of infected aerosols by a suspicious person during speech or breath [18]. Numerous experimental studies have found that the SARS-CoV-2 RNA could survive in the air from 3 to 16 h. However, results generated from studies performed in true healthcare facilities remain debatable [19, 20].

### Fomite transmission

Fomites are also known as contaminated surfaces containing traces of any infectious agent. Various studies found detectable traces of SARS-CoV-2 RNA on the surfaces used by infected patients. Although, there are no specific reports directly demonstrating fomite transmission, this mode of infection remained a possible way of transmission in a similar type of infection reported earlier [19, 21, 22].

### Biological samples

Biological samples include urine and feces. Although SARS-CoV-2 has been found in the patient’s urine and feces samples, no report confirmed them as a possible transmission route [23].

### Blood serum and plasma and blood transfusion

Even though the SARS-CoV-2 has been found in patients’ blood serum and plasma, lower SARS-CoV-2 viral titers suggest a minor risk of viral spread through serum and plasma [23, 24].

### Breastfeeding

Although the SARS-CoV-2 RNA was found in few breast milk samples of infected mothers, there is no evidence that this virus will be able to reach target sites in infants to start replication. Until this day, WHO recommended breastfeeding to be continued in infants by the mothers who are suspected or infected with SARS-CoV-2 [24, 25].

### Human to pet animals/livestock and vice versa

It is unclear whether the pets are able to transmit and infect SARS-CoV-2 in humans; however, there could be a zoonotic possibility of transmission [26]. In a recent study, an infected cat was able to transmit the SARS-CoV-2 virus to other cats living in the same cage. There are few studies showing that infected humans can transmit the virus and infect other animals including dogs, cats and farmed mink. Till date, there is no evidence of the SARS-CoV-2 transmission from humans to horses or livestock or vice versa. Detailed information about SARS-CoV-2 spread through animals can be found at the website of the College of Veterinary Medicine, The Ohio State University (https://vet.osu.edu/about-us/news/covid-19-and-animals#Horses%20Livestock%20risk%20to%20humans) and other online sources.

## Possible infection spread scenarios/risk factors/prevention

WHO has recently published a scientific brief describing when a person may be able to spread the virus (https://www.who.int/news-room/commentaries/detail/transmission-of-sars-cov-2-implications-for-infection-prevention-precautions)? Briefly, the virus can be detected in people 1–3 days before the appearance of any symptom with high viral loads at early onset. The viral loads will start to decline gradually. However, a positive PCR report does not mean a person is infected and will be able to transmit the disease. It might take days to detect the viable virus during the early or mild stage.

### Close contacts

Nevertheless, nearby and prolonged contact was found to be a main reason for the SARS-CoV-2 transmission. There are more chances of spreading the virus between family members if proper measures are not taken soon after the detection of onset infection in one of the family or close contact member. Moreover, the close contact outside at various places like cinema, meal sharing, gym, praying, or workplace increases the risk of virus transmission [27–29].

### Asymptomatic contacts

It is also possible for a person without symptoms to transmit the virus to the healthy ones. It is important to know that which person is virus infected without showing any symptoms. Recent studies from the globe revealed that approximately 16–23% of asymptomatic persons did not develop the symptoms at all, and this range could be up to 41%. The most furious part is the recent research showing that there is a 44% (25–69%) chance that the viral transmission may have occurred before any symptom appearance. Although it is difficult to trace infection before symptoms appear, early testing and tracing would help to minimize the virus transmission [29–31].

### Late SARS-CoV—diagnosis and quarantine

All the available data show that reducing or limiting the close contacts is the most effective way to reduce the transmission of the virus. Moreover, the early and timely diagnosis will help to trace the possible infection whereas self or enforced quarantine will minimize the spread to any secondary contact [32]. WHO has published a detailed guidance document for possible quarantine times that usually range from 5 to 6 days to 2 weeks (https://www.who.int/publications/i/item/considerations-for-quarantine-of-individuals-in-the-context-of-containment-for-coronavirus-disease-(covid-19)?

### Avoiding social gatherings

As there are significant chances to contact with the asymptomatic persons during social gatherings in or outside or at workplaces, it is advised to minimize such gatherings or take necessary measures before attending such. Personal protective measures such as masks and gloves, and maintaining hygiene can reduce the chance of virus transmission. The use of antimicrobial agents and minimum required force at workplaces or the option to work from home wherever possible will reduce the chances of viral spread [32, 33].

### Abiding by community regulations

As most of the SARS-CoV-2 infections spread through traveling of the infected persons from one place to another, it is recommended to abide by all the laws and regulations advised by the communities or countries to reduce the chances of virus spread in non-infected areas or reduce infection rates in supervised communities [32–34].

## Persons diagnosed/pre-infected with other diseases

Like other diseases, it is possible that some abnormalities or pre-existing diseases could be the possible risk factors for SARS-CoV-2 infection or vice versa. Unfortunately, there are few studies that addressed this type of risk factor [35–39]. A summary of these coincidences has been given below.

### SARS-CoV-2 and cancer

Cancer patients were found to be at increased risk of the SARS-CoV-2 infection and having an increased death rate. A recent study by Lee et al. [40] found that the susceptibility to SARS-CoV-2 infection is different among patients with different types of tumors. The UK coronavirus monitoring project (UKCCMP) and other researchers found that the patients with hematological cancers like leukemia, lymphoma, or myeloma were more vulnerable to SARS-CoV-2 than other forms of cancers [41–43].

### Patients with diabetes or associated diseases

According to a report from webmed.com (https://www.webmd.com/diabetes/diabetes-and-coronavirus#:~:text=Early%20studies%20have%20shown%20that,to%20die%20from%20the%20virus), about 25% of patients with severe COVID-19 symptoms had diabetes, and infection might increase the pre-existed complexities in the diabetic patients. This risk could be increased in the presence of other diseases like lung or heart. As per guidelines/recommendations from the American Diabetes Association, there is not enough data showing that diabetic persons are more likely to be infected with the SARS-CoV-2. However, diabetes itself worsened the condition in case of infection (https://www.diabetes.org/coronavirus-covid-19/how-coronavirus-impacts-people-with-diabetes). Similar findings were observed from the cohort-based studies across the globe [36–38, 43]. In a recent study by Dennis et al. [35] in England found that the type-II diabetes increased the risk of fatality in persons infected with SARS-CoV-2, especially in younger patients. It has also been noted that the hyperglycemia not only enhanced the SARS-CoV-2 replication in monocytes but also increased the ACE2 expression. Moreover, monocytes taken from diabetic patients were more vulnerable to SARS-CoV-2 than normal [44].

### Patients with neuro disorders

There are few studies describing any possible association of the SARS-CoV-2 infection with neurological disorders, including dementia and Alzheimer’s disease [45–48]. It has been found that the cerebral white matter is most susceptible to SARS-CoV-2 and might increase cognitive dysfunction [45, 49]. There is emerging evidence that the SARS-CoV-2 infection might induce amyloid beta and tau toxicity in the patients with Alzheimer’s and associated dementia diseases. This could be due to the depleted ACE2 and associated RAS activation. Moreover, there is strong evidence that the ACE2 depletion in APOE 4 individuals increased the cognitive decline [46, 50]. It is also possible that SARS-CoV-2 might reduce immune response and increase the death rate in people already having dementia and worsen psychiatric symptoms and behavioral disturbances [51].

### SARS-CoV-2 and kidney disease

It is interesting to note that the SARS-CoV-2 was found in autopsy samples of the COVID-19 patients whether they had pre-kidney problems or not [52]. The same study also found that the highest copies of the SARS-CoV-2 per cell were detected in the respiratory tract, whereas lower viral loads were detected in the kidneys, liver, heart, brain, and blood. In silico analysis showed that kidneys have high levels of RNA expression responsible for ACE2, TMPRSS2, and cathepsin L that might in turn induce the SARS-CoV-2 susceptibility for kidneys. Meanwhile, vulnerability of the podocytes and tubular epithelial cells to SARS-CoV-2 infection was expected to induce the chances of renal injury [53]. It is evident from various studies that the SARS-CoV-2 infection induces acute kidney injury that might result in disease severity and premature death within the first 3 weeks of infection [54].

### SARS-CoV-2 and liver injury

It has been found that the SARS-CoV-2 infection to the liver cells is responsible for liver injury [55]. There is evidence that high expression of ACE2 receptors in the liver cells might induce SARS-CoV-2-associated injury in infected patients [55]. The other possibility could be an induced inflammatory response (increased cytokines levels) after infection that may lead to liver injury [56]. Increased levels of liver enzymes and biochemical markers, including AST, ALT, and bilirubin were observed in patients with the SARS-CoV-2 infection. However, there are no reports about liver failure in patients with COVID-19 [57].

### Cardiac complications in SARS-CoV-2 infected patients

The virus was detected in the cardiac myocardium autopsy cases of the patients infected with SARS-CoV-2 with a significant viral replication ability. Moreover, induced cytokine expression in the patients with high virus copies suggests that cytokine-induced organ dysfunction may worsen the disease [58]. Meanwhile, in multiple clinical trials, results reviewed by Wu et al. and Dhakal et al. also found higher ACE2 expression, hypoxemia, cytokine storm, stress, and cardiotoxicity of the anti-viral drugs in patients with COVID-19 symptoms [59, 60]. Overall, SARS-CoV-2 infection in patients with pre-cardiovascular disease could contribute to high mortality rates [61].

### Ophthalmic (eye) manifestation of SARS-CoV-2

SARS-CoV-2 has strong affection with the ACE2 receptors. Lower levels of ACE2 and TMPRSS2 were found in the human cornea and conjunctival tissues when compared to the heart and lungs [62, 63]. A recent review by Perez-Bartolome et al. on clinical cases and other reports regarding the potential ocular manifestations of SARS-CoV-2 [64] described that virus infection could result in eye redness, together with erythroderma and fever. Besides this, there are chances that the SARS-CoV-2 could be present in tears from infected patients. In a recent case report, the tear samples collected from some of the patients were positive for COVID-19 presence [65]. Although there are no indications that the viral infection could lead to blindness, a recent case report showed that a COVID-19-positive patient with reversible encephalopathy syndrome got persistent cortical blindness.

Overall, these observations not only demand high care of the corona-infected patients but also the persons taking care of them, including the medical staff and family members. All of them should regularly test themselves and take all necessary protective measures.

## Current epidemiology and economic situation

### SARS-CoV-2 epidemiology

The World Health Organization is publishing a weekly global report on the current epidemiology of the COVID-19 infection that can be accessed through https://www.who.int/publications/m. On this day of the 24th of Nov 2021, 25.6 million cases were reported that were 15% low when compared to the previous week. There was also around a 3% decline in the number of deaths due to coronavirus. Until now, there are 259,502,031 cumulative cases with 5,183,003 deaths globally with a death rate of 2.0%. Table 1 adopted from WHO is reporting the current global scenario showing a decline in infection as well as death rates worldwide. Despite “WHO” worldometer (https://www.worldometers.info/coronavirus/) is also updating the COVID-19 infections globally on a daily basis. According to worldometer, there are 261,036,742 coronavirus cases with 5,209,580 deaths globally on the 24th of Nov 2021. The recovered cases were 235,860,474 whereas a total of 19,966,688 were active cases with 83,223 in the serious or critical stage.

**Table 1:** Recent reported and cumulative COVID-19 cases and deaths, by WHO regions and globally

WHO region	New cases in last 7 days (%)	Change in new cases in last 7 days*	Cumulative cases (%)	New deaths in last 7 days (%)	Change in new deaths in last 7 days*	Cumulative deaths (%)
**Americas**	1315480 (48%)	− 16%	48228712 (45%)	44385 (55%)	− 2%	1136906 (48%)
**Europe**	968943 (36%)	− 18%	36575529 (34%)	28404 (35%)	− 19%	812410 (34%)
**Southeast Asia**	154414 (6%)	− 13%	13188211 (12%)	2340 (3%)	− 9%	202607 (8%)
**Eastern Mediterranean**	170445 (6%)	7%	5998998 (6%)	2519 (3%)	− 9%	139468 (6%)
**Africa**	68115 (2%)	− 20%	2723431 (3%)	2558 (3%)	− 21%	68294 (3%)
**Western Pacific**	49577 (2%)	− 20%	1531366 (1%)	1134 (1%)	− 13%	27019 (1%)
**Global**	**2726974 (100%)**	**− 16%**	**108246992 (100%)**	**81340 (100%)**	**− 10%**	**2386717 (100%)**

### SARS-CoV-2, global economy, and disease spread

To ensure better treatment and a healthy environment, there should be a strong economy to provide funding to cope with this type of pandemic. A good economy resulted in more resources to deploy for scientific research and development. It has been obvious that most governments misinterpreted the risks of SARS-CoV-2 infection from the beginning of the pandemic and were not ready to act properly [66]. The intensive SARS-CoV-2 breakdown resulted in a sudden loss of global economies due to the long-lasting lockdowns, closure of multiple businesses, reduced productivity, excess labor, and reduced working hours. According to BBC, there was approximately − 15 to − 5% loss of economic growth in most of the countries whereas the global economy shrunk by 4.4% in 2020 worst since the great depression of the 1930s (https://www.bbc.com/news/business-51706225).

## Vaccination(s) against SARS-CoV-2 infection

Soon after the rise of the pandemic, multiple labs started the process to create any effective vaccination against the SARS-CoV-2 infection. More than 200 vaccine candidates were reported to be involved in vaccination development (https://www.who.int/publications/m/item/draft-landscape-of-covid-19-candidate-vaccines). According to this report, there are 69 vaccines in clinical development while 181 in pre-clinical development. There are 33% protein subunit-based vaccines while 14% each non-replicating viral vector, DNA, and inactivated virus vaccines. The remaining vaccines might contain replicating viral vector or virus-like particles or some combinations of the abovementioned products. Till date, a total of 2,156,384,616 vaccine doses have been administered (WHO: https://covid19.who.int/). In Table 2, we have mentioned the vaccines currently in phase 3 or available in the market now for the immunization.

**Table 2:** Details of vaccines against SARS-CoV-2 currently available or in clinical phase 3

No.	Platform	Type	Doses	Dose schedule	Route	Developers
**1**	Inactivated virus	SARS-CoV-2 vaccine (inactivated)	2	Day 0 + 14	IM	Sinovac Research and Development Co., Ltd
**2**	Inactivated virus	Inactivated SARS-CoV-2 vaccine (Vero cell)	2	Day 0 + 21	IM	Sinopharm + China National Biotec Group Co + Wuhan Institute of Biological Products
**3**	Inactivated virus	Inactivated SARS-CoV-2 vaccine (Vero cell)	2	Day 0 + 21	IM	Sinopharm + China National Biotec Group Co + Beijing Institute of Biological Products
**4**	Viral vector (non-replicating)	ChAdOx1-S—(AZD1222) (Covishield)	1–2	Day 0 + 28	IM	AstraZeneca + University of Oxford
**5**	Viral vector (non-replicating)	Recombinant novel coronavirus vaccine (adenovirus type 5 vector)	1	Day 0	IM	CanSino Biological Inc./Beijing Institute of Biotechnology
**6**	Viral vector (non-replicating)	Gam-COVID-Vac adeno-based (rAd26-S+rAd5-S)	2	Day 0 + 21	IM	Gamaleya Research Institute ; Health Ministry of the Russian Federation
**7**	Viral vector (non-replicating)	Ad26.COV2.S	1-2	Day 0 or Day 0 +56	IM	Janssen Pharmaceutical
**8**	Protein subunit	SARS-CoV-2 rS/Matrix M1-adjuvant (full-length recombinant SARS CoV-2 glycoprotein nanoparticle vaccine adjuvanted with Matrix M)	2	Day 0 + 21	IM	Novavax
**9**	RNA-based vaccine	mRNA-1273	2	Day 0 + 28	IM	Moderna + National Institute of Allergy and Infectious Diseases (NIAID)
**10**	RNA -based vaccine	BNT162 (3 LNP-mRNAs )	2	Day 0 + 21	IM	Pfizer/BioNTech + Fosun Pharma
**11**	Protein subunit	Recombinant SARS-CoV-2 vaccine (CHO cell)	2-3	Day 0 + 28 or Day 0 + 28 + 56	IM	Anhui Zhifei Longcom Biopharmaceutical + Institute of Microbiology, Chinese Academy of Sciences
**12**	RNA-based vaccine	CVNCOV vaccine	2	Day 0 + 28	IM	CureVac AG
**13**	Inactivated virus	SARS-CoV-2 vaccine (vero cells)	2	Day 0 + 28	IM	Institute of Medical Biology + Chinese Academy of Medical Sciences
**14**	Inactivated virus	QazCovid-in®—COVID-19 inactivated vaccine	2	Day 0 + 21	IM	Research Institute for Biological 15Safety Problems, Rep of Kazakhstan
**15**	DNA-based vaccine	INO-4800 + electroporation	2	Day 0 + 28	ID	Inovio Pharmaceuticals + International Vaccine Institute + Advaccine (Suzhou) Biopharmaceutical Co., Ltd
**16**	DNA-based vaccine	AG0301-COVID19	2	Day 0 + 14	IM	AnGes + Takara Bio + Osaka University
**17**	DNA-based vaccine	nCov vaccine	3	Day 0 + 28 + 56	ID	Zydus Cadila
**18**	DNA-based vaccine	GX-19	2	Day 0 + 28	IM	Genexine Consortium
**19**	Inactivated virus	Whole-virion inactivated SARS-CoV-2 vaccine (BBV152)	2	Day 0 + 14	IM	Bharat Biotech International Limited
**20**	Protein subunit	SCB-2019 + AS03 or CpG 1018 adjuvant plus alum adjuvant (native like trimeric subunit spike protein vaccine)	2	Day 0 + 21	IM	Clover Biopharmaceuticals Inc./GSK/Dynavax
**21**	Protein subunit	UB-612 (Multitope peptide based S1-RBD-protein based vaccine)	2	Day 0+28	IM	COVAXX + United Biomedical Inc
**22**	Virus-like particle	Coronavirus-like particle COVID-19 (CoVLP)	2	Day 0 + 21	IM	Medicago Inc.

## Candidate drugs against SARS-CoV-2 infection

Although multiple vaccines against the SARS-CoV-2 infection are currently in the clinical stage or available in a limited amount for people, the requirement of the drug against any infection remains necessary. Unfortunately, till date, there is no effective drug against the SARS-CoV-2. There are two types of drug candidates which might help to cope with this deadly infection in the future: (i) blocking of virus entry to host and (ii) inhibition of virus replication [67]. Below is a brief outline about these candidate drugs.

### Inhibiting virus entry to host

Currently, there are two known virus-host interaction sites/targets in humans namely TMPRSS2 serine protease and ACE2 receptors [68]. TMPRSS2 is involved in the cleavage and activation of the SARS-CoV-2 spike protein whereas ACE2 is required for the SARS-CoV-2 entry to the human cells. TMPRSS2 inhibition in the human cell lines showed a reduction in SARS-CoV-2 infection [68]. Camostat mseilate (N,N-dimethylcarbamoylmethyl 4-(4-guanidinobenzoyloxy)-phenylacetate) [39] and Nafamostat mesylate (6-amidino-2-naphthyl-4-guanidino benzoate-dimethanesulfonate) [69] are potent inhibitors of TMPRSS2 and are under clinical trials against the SARS-CoV-2 infection.

The other important target to reduce the virus entry in humans is the inhibition of human ACE2 receptors. The compounds and drugs that showed ACE2 inhibition included chloroquine [70] and hydroxychloroquine [71], cephranthine [72], ivermectin [73], selamectin, and mefloquine hydrochloride [74]. These drugs were previously used to treat Q-fever and malaria and as prophylaxis and have been known as anti-helminthic, parasiticide, and anti-viral. Besides these drugs, there are some drugs in experimental trials, including synthetic DX600, MLN-4760, and TAPI-2 [50]. There are some plant-derived compounds that may inhibit ACE2 and in turn block SARS-CoV-2 entry into the host cells/ human [75].

### Anti- viral drugs

Other proposed drugs are based on the inhibition of viral replication and assembly in the host cells. These include remdesivir, lopinavir, umifenovir, favipiravir, arbidol, ribavirin, sofosbuvir, ritonavir, nelfinavir, and dolutegravir. Although these drugs showed promising results in the human cell cultures in the laboratory, the outcome in actual patients was not so promising. However, recent clinical trials showed that different combinations of these drugs with others could be a good therapeutic option in the future [67, 76].

### Antibody neutralization

Another proposed therapy to deal with the SARS-CoV-2 infection was to inject the already recovered (convalescent) patient’s plasma into the diseased person [70, 76, 77]. The current clinical trials have not shown any significant improvement in the infected persons after plasma therapy; however, the development of target-specific monoclonal antibodies against the SARS-CoV-2 spike proteins is underway.

### Natural compounds and immune therapy

Alongside these synthetic drugs, many natural products and their derivatives like plant-based steroids and phytochemicals, including flavonoids, seemed to be effective against various infections in the past [75]. These products not only were able to reduce the virus entry and infection but also were able to boost the immune response. Multiple studies have found that the SARS-CoV-2 infection might result in excessive inflammation and uncontrolled cytokine storm that could lead to severe disease complexities and patient death [78].

### Other options

Unfortunately, the available drugs for the TMRSS2 and ACE2 inhibition or SARS-CoV-2-specific anti-viral drugs did not show promising results in reducing SARS-CoV-2 infection, and there is a need to develop some disease-specific drugs. It has been an established fact that certain drugs have serious limitations with multiple side effects. Based on previous findings, in silico results showed that inhibiting ACE2 phosphorylation at Ser-787 through O-β-GlcNAcylation has the potential not only to reduce viral-host binding but also virus entry in host cells [15]. As persons with pre-diabetes and neurological disorders like dementia are highly vulnerable to SARS-CoV-2 infection [35–38, 41, 44, 47–51], it could be possible that drugs used to treat lower blood glucose levels and reduce neurological symptoms might be helpful to reduce the severity of SARS-CoV-2 infection [79–82]. Other drugs that fall into this category could be tested for the SARS-CoV-2 infection severity. Moreover, multiple genes, metabolites, proteins, and extracellular RNAs were found to be associated with several clinical parameters during SARS-CoV-2 infection. These genes or proteins have been suggested as possible biomarkers and any change in their expressions could affect the severity of the SARS-CoV-2 infection as observed in other diseases like dementia and cancer [82–84].

## Future perspective only

So far, understanding of the SARS-CoV-2 structure and its interaction with the host and the currently available vaccines and drugs helped to decrease the number of growing infection cases and deaths suffering from the SARS-CoV-2 infection. However, the current emergence of the SARS-CoV-2 variants (https://www.who.int/csr/don/31-december-2020-sars-cov2-variants/en/) like SARS-CoV-2 D614G (a mutation in the spike protein), SARS-CoV-2 VOC 202012/01, SARS-CoV-2 N501Y, Alpha (B.1.1.7, +S:484K/ +S:452K), Beta (B.1.351, +S:L18F), Gamma (P.1, +S:681H), Delta (B.1.617.2, +S:417N/ +S:484K)  and SARS-CoV-2 Omicron (B.1.1.529, +S:R346K) could be a potential threat against the global cumulative efforts to combat this deadly disease (https://www.who.int/en/activities/tracking-SARS-CoV-2-variants/).

## Conclusions

Unfortunately, till date, there are no specific drugs or efficient vaccines to treat the SARS-CoV-2 infection. The current available vaccines are under trial or still under data collection from the immunized persons. Apparently, it would take a long time to assess the efficacy of the current available vaccines in immunized persons. Although vaccination is a potent way to reduce infection via immunity development, efforts should be increased in developing target-specific drugs to combat the SARS-CoV-2 in the future. Moreover, societies/communities should be properly informed about the dangers of this deadly disease, and governments should implicate such laws that may be helpful in reducing corona infections without lowering the living standards of ordinary persons.

## Data Availability

Not applicable

## References

[1] Okada P, Buathong R, Phuygun S et al (2020) Early transmission patterns of coronavirus disease 2019 (COVID-19) in travellers from Wuhan to Thailand, January 2020. Euro Surveill 25(8). 10.2807/1560-7917.ES.2020.25.8.2000097 10.2807/1560-7917.ES.2020.25.8.2000097 PMC7055038 32127124

[2] Zhou P, Yang XL, Wang XG (2020). A pneumonia outbreak associated with a new coronavirus of probable bat origin. Nature.

[3] Kadam SB, Sukhramani GS, Bishnoi P, Pable AA, Barvkar VT (2021). SARS-CoV-2, the pandemic coronavirus: molecular and structural insights. J Basic Microbiol.

[4] Walls AC, Park YJ, Tortorici MA, Wall A, McGuire AT, Veesler D (2020). Structure, function, and antigenicity of the SARS-CoV-2 spike glycoprotein. Cell.

[5] Tortorici MA, Veesler D (2019). Structural insights into coronavirus entry. Adv Virus Res.

[6] Park JE, Li K, Barlan A (2016). Proteolytic processing of Middle East respiratory syndrome coronavirus spikes expands virus tropism. Proc Natl Acad Sci USA.

[7] Walls AC, Tortorici MA, Bosch BJ (2016). Cryo-electron microscopy structure of a coronavirus spike glycoprotein trimer. Nature.

[8] Juraszek J, Rutten L, Blokland S (2021). Stabilizing the closed SARS-CoV-2 spike trimer. Nat Commun.

[9] Zhou T, Tsybovsky Y, Gorman J (2020). Cryo-EM structures of SARS-CoV-2 spike without and with ACE2 reveal a pH-dependent switch to mediate endosomal positioning of receptor-binding domains. Cell Host Microbe.

[10] Bangaru S, Ozorowski G, Turner HL (2020). Structural analysis of full-length SARS-CoV-2 spike protein from an advanced vaccine candidate. Science.

[11] Barnes CO, Jette CA, Abernathy ME (2020). SARS-CoV-2 neutralizing antibody structures inform therapeutic strategies. Nature.

[12] Anand SP, Chen Y, Prévost J (2020). Interaction of human ACE2 to membrane-bound SARS-CoV-1 and SARS-CoV-2 S glycoproteins. Viruses.

[13] Shang J, Wan Y, Luo C (2020). Cell entry mechanisms of SARS-CoV-2. Proc Natl Acad Sci USA.

[14] Li F, Li W, Farzan M, Harrison SC (2005). Structure of SARS coronavirus spike receptor-binding domain complexed with receptor. Science.

[15] Ahmad W, Shabbiri K, Islam N (2020) O-β-GlcNAcylation, chloroquine and 2-hydroxybenzohydrazine may hamper SARS-CoV-2 entry to human via inhibition of ACE2 phosphorylation at Ser787 but also induce disruption of virus-ACE2 binding. Preprint 10.20944/preprints202004.0390.v1

[16] Li S, Li S, Disoma C et al (2020) SARS-CoV-2: Mechanism of infection and emerging technologies for future prospects. Rev Med Virol. 10.1002/rmv.2168 10.1002/rmv.2168 35349206

[17] (2014) Infection prevention and control of epidemic-and pandemic-prone acute respiratory infections in health care. World Health Organization, Geneva. Available at https://apps.who.int/iris/bitstream/handle/10665/112656/9789241507134_eng.pdf;jsessionid=41AA684FB64571CE8D8A453C4F2B2096?sequence=1. Accessed 20-22 Oct 2021. 24983124

[18] Bourouiba L (2020). Turbulent gas clouds and respiratory pathogen emissions: potential implications for reducing transmission of COVID-19. JAMA..

[19] Van Doremalen N, Bushmaker T, Morris DH, Holbrook MG, Gamble A, Williamson BN (2020). Aerosol and surface stability of SARS-CoV-2 as compared with SARS-CoV-1. N Engl J Med.

[20] Fears AC, Klimstra WB, Duprex P, Weaver SC, Plante JA, Aguilar PV et al (2020) Persistence of severe acute respiratory syndrome coronavirus 2 in aerosol suspensions. Emerg Infect Dis 26(9). 10.3201/eid2609.201806 10.3201/eid2609.201806 PMC7454081 32568661

[21] Chia PY, for the Singapore Novel Coronavirus Outbreak Research T, Coleman KK, Tan YK, SWX O, Gum M et al (2020) Detection of air and surface contamination by SARS-CoV-2 in hospital rooms of infected patients. Nat Commun 11(1). 10.1038/s41467-020-16670-2 10.1038/s41467-020-16670-2 PMC7260225 32472043

[22] Liu Y, Ning Z, Chen Y, Guo M, Liu Y, Gali NK (2020). Aerodynamic analysis of SARS-CoV-2 in two Wuhan hospitals. Nature..

[23] Wang W, Xu Y, Gao R, Lu R, Han K, Wu G (2020). Detection of SARS-CoV-2 in different types of clinical specimens. JAMA..

[24] Chang L, Zhao L, Gong H, Wang L, Wang L (2020). Severe acute respiratory syndrome coronavirus 2 RNA detected in blood donations. Emerg Infect Dis.

[25] Breastfeeding and COVID-19. World Health Organization, Geneva. 2020. Available at https://www.who.int/newsroom/commentaries/detail/breastfeeding-and-covid-19. Accessed 20-22 Oct 2021.

[26] Swelum AA, Shafi ME, Albaqami NM (2020). COVID-19 in Human, Animal, and Environment: A Review. Front Vet Sci.

[27] Davies N, Klepac P, Liu Y, Prem K, Jit M, CCMID COVID-19 Working Group et al (2020) Age-dependent effects in the transmission and control of COVID-19 epidemics. Nat Med. 10.1038/s41591-020-0962-9 10.1038/s41591-020-0962-9 32546824

[28] Qian G, Yang N, Ma AHY, Wang L, Li G, Chen X et al (2020) COVID-19 Transmission Within a Family Cluster by Presymptomatic Carriers in China. Clin Infect Dis. 10.1093/cid/ciaa316 10.1093/cid/ciaa316 PMC7184331 32201889

[29] Kimball A, Hatfield KM, Arons M, James A, Taylor J, Spicer K (2020). Asymptomatic and presymptomatic SARS-CoV-2 infections in residents of a long-term care skilled nursing facility—King County, Washington, March 2020. MMWR Surveill Summ.

[30] Wang Y, Liu Y, Liu L, Wang X, Luo N, Ling L (2020). Clinical outcome of 55 asymptomatic cases at the time of hospital admission infected with SARS-Coronavirus-2 in Shenzhen, China. J Infect Dis.

[31] Sakurai A, Sasaki T, Kato S, Hayashi M, Tsuzuki S-I, Ishihara T et al (2020) Natural history of asymptomatic SARS-CoV-2 infection. N Engl J Med. 10.1056/NEJMc2013020 10.1056/NEJMc2013020 PMC7304419 32530584

[32] Kevadiya BD, Machhi J, Herskovitz J (2021). Diagnostics for SARS-CoV-2 infections. Nat Mater.

[33] Wadman M, Couzin-Frankel J, Kaiser J, Matacic C (2020). A rampage through the body. Science..

[34] Pan Y (2020). Serological immunochromatographic approach in diagnosis with SARS-CoV-2 infected COVID-19 patients. J Infect.

[35] Dennis JM, Mateen BA, Sonabend R (2021). Type 2 diabetes and COVID-19-Related mortality in the critical care setting: a national cohort study in England, March-July 2020. Diabetes Care.

[36] Drucker DJ (2021). Diabetes, obesity, metabolism, and SARS-CoV-2 infection: the end of the beginning. Cell Metab.

[37] You JH, Lee SA, Chun SY (2020). Clinical outcomes of COVID-19 patients with type 2 diabetes: a population-based study in Korea. Endocrinol Metab (Seoul).

[38] Orioli L, Servais T, Belkhir L (2021). Clinical characteristics and short-term prognosis of in-patients with diabetes and COVID-19: a retrospective study from an academic center in Belgium. Diabetes Metab Syndr.

[39] Breining P, Frølund AL, Højen JF (2021). Camostat mesylate against SARS-CoV-2 and COVID-19-rationale, dosing and safety. Basic Clin Pharmacol Toxicol.

[40] Lee LYW, Cazier JB, Starkey T (2020). COVID-19 prevalence and mortality in patients with cancer and the effect of primary tumour subtype and patient demographics: a prospective cohort study. Lancet Oncol.

[41] Yang K, Sheng Y, Huang C (2020). Clinical characteristics, outcomes, and risk factors for mortality in patients with cancer and COVID-19 in Hubei, China: a multicentre, retrospective, cohort study. Lancet Oncol.

[42] Kuderer NM, Choueiri TK, Shah DP (2020). Clinical impact of COVID-19 on patients with cancer (CCC19): a cohort study. Lancet.

[43] Dai M, Liu D, Liu M (2020). Patients with cancer appear more vulnerable to SARS-CoV-2: a multicenter study during the COVID-19 outbreak. Cancer Discov.

[44] Codo AC, Davanzo GG, Monteiro LB (2020). Elevated glucose levels favor SARS-CoV-2 infection and monocyte response through a HIF-1α/glycolysis-dependent axis. Cell Metab.

[45] Miners S, Kehoe PG, Love S (2020). Cognitive impact of COVID-19: looking beyond the short term. Alzheimers Res Ther.

[46] Lemprière S (2020). SARS-CoV-2 and the brain to be studied long-term. Nat Rev Neurol.

[47] Ellul MA, Benjamin L, Singh B (2020). Neurological associations of COVID-19. Lancet Neurol.

[48] Ahmed MU, Hanif M, Ali MJ (2020). Neurological manifestations of COVID-19 (SARS-CoV-2): a review. Front Neurol.

[49] Acharya A, Kevadiya BD, Gendelman HE, Byrareddy SN (2020). SARS-CoV-2 infection leads to neurological dysfunction. J NeuroImmune Pharmacol.

[50] Abate G, Memo M, Uberti D (2020). Impact of COVID-19 on Alzheimer’s disease risk: viewpoint for research action. Healthcare (Basel).

[51] Numbers K, Brodaty H (2021). The effects of the COVID-19 pandemic on people with dementia. Nat Rev Neurol.

[52] Puelles VG, Lütgehetmann M, Lindenmeyer MT (2020). Multiorgan and renal tropism of SARS-CoV-2. N Engl J Med.

[53] Martinez-Rojas MA, Vega-Vega O, Bobadilla NA (2020). Is the kidney a target of SARS-CoV-2?. Am J Physiol Ren Physiol.

[54] Braun F, Lütgehetmann M, Pfefferle S (2020). SARS-CoV-2 renal tropism associates with acute kidney injury. Lancet.

[55] Zhang C, Shi L, Wang FS (2020). Liver injury in COVID-19: management and challenges. Lancet Gastroenterol Hepatol.

[56] Wu J, Song S, Cao HC, Li LJ (2020). Liver diseases in COVID-19: etiology, treatment and prognosis. World J Gastroenterol.

[57] Lozano-Sepulveda SA, Galan-Huerta K, Martínez-Acuña N, Arellanos-Soto D, Rivas-Estilla AM (2020). SARS-CoV-2 another kind of liver aggressor, how does it do that?. Ann Hepatol.

[58] Lindner D, Fitzek A, Bräuninger H (2020). Association of cardiac infection With SARS-CoV-2 in confirmed COVID-19 autopsy cases. JAMA Cardiol.

[59] Wu L, O’Kane AM, Peng H, Bi Y, Motriuk-Smith D, Ren J (2020). SARS-CoV-2 and cardiovascular complications: from molecular mechanisms to pharmaceutical management. Biochem Pharmacol.

[60] Dhakal BP, Sweitzer NK, Indik JH, Acharya D, William P (2020). SARS-CoV-2 infection and cardiovascular disease: COVID-19 heart. Heart Lung Circ.

[61] Nishiga M, Wang DW, Han Y, Lewis DB, Wu JC (2020). COVID-19 and cardiovascular disease: from basic mechanisms to clinical perspectives. Nat Rev Cardiol.

[62] Ma D, Chen CB, Jhanji V (2020). Expression of SARS-CoV-2 receptor ACE2 and TMPRSS2 in human primary conjunctival and pterygium cell lines and in mouse cornea. Eye (Lond).

[63] Chen X, Yu H, Mei T et al (2020) SARS-CoV-2 on the ocular surface: is it truly a novel transmission route? Br J Ophthalmol:bjophthalmol-2020-316263. 10.1136/bjophthalmol-2020-316263 10.1136/bjophthalmol-2020-316263 PMC8380887 32788324

[64] Pérez-Bartolomé F, Sánchez-Quirós J (2021). Ocular manifestations of SARS-CoV-2: literature review. Arch Soc Esp Oftalmol (Engl Ed).

[65] Loon SC, Teoh SC, Oon LL (2004). The severe acute respiratory syndrome coronavirus in tears. Br J Ophthalmol.

[66] Pak A, Adegboye OA, Adekunle AI, Rahman KM, McBryde ES, Eisen DP (2020). Economic consequences of the COVID-19 outbreak: the need for epidemic preparedness. Front Public Health.

[67] McKee DL, Sternberg A, Stange U, Laufer S, Naujokat C (2020). Candidate drugs against SARS-CoV-2 and COVID-19. Pharmacol Res.

[68] Hoffmann M, Kleine-Weber H, Schroeder S (2020). SARS-CoV-2 cell entry depends on ACE2 and TMPRSS2 and is blocked by a clinically proven protease inhibitor. Cell.

[69] Hoffmann M, Schroeder S, Kleine-Weber H, Müller MA, Drosten C, Pöhlmann S (2020). Nafamostat mesylate blocks activation of SARS-CoV-2: new treatment option for COVID-19. Antimicrob Agents Chemother.

[70] Stahlmann R, Lode H (2020). Medication for COVID-19-an overview of approaches currently under study. Dtsch Arztebl Int.

[71] Yao X, Ye F, Zhang M (2020). In vitro antiviral activity and projection of optimized dosing design of hydroxychloroquine for the treatment of severe acute respiratory syndrome coronavirus 2 (SARS-CoV-2). Clin Infect Dis.

[72] Rogosnitzky M, Okediji P, Koman I (2020). Cepharanthine: a review of the antiviral potential of a Japanese-approved alopecia drug in COVID-19. Pharmacol Rep.

[73] Sharun K, Dhama K, Patel SK (2020). Ivermectin, a new candidate therapeutic against SARS-CoV-2/COVID-19. Ann Clin Microbiol Antimicrob.

[74] Fan HH, Wang LQ, Liu WL (2020). Repurposing of clinically approved drugs for treatment of coronavirus disease 2019 in a 2019-novel coronavirus-related coronavirus model. Chin Med J.

[75] Verma S, Twilley D, Esmear T (2020). Anti-SARS-CoV natural products with the Potential to inhibit SARS-CoV-2 (COVID-19). Front Pharmacol.

[76] Valle C, Martin B, Touret F (2020). Drugs against SARS-CoV-2: what do we know about their mode of action?. Rev Med Virol.

[77] Zhou G, Zhao Q (2020). Perspectives on therapeutic neutralizing antibodies against the novel coronavirus SARS-CoV-2. Int J Biol Sci.

[78] Yuan C, Li R, Liu G, Pan Y (2021). Potential of immune-related therapy in COVID-19. Front Pharmacol.

[79] Ahmad W, Ebert PR (2018). 5-Methoxyindole-2-carboxylic acid (MICA) suppresses Aβ-mediated pathology in C. elegans. Exp Gerontol.

[80] Ahmad W (2018). Dihydrolipoamide dehydrogenase suppression induces human tau phosphorylation by increasing whole body glucose levels in a C. elegans model of Alzheimer’s disease. Exp Brain Res.

[81] Ahmad W, Ebert PR (2017). Metformin attenuates Aβ pathology mediated through levamisole sensitive nicotinic acetylcholine receptors in a C. elegans model of Alzheimer’s disease. Mol Neurobiol.

[82] Ahmad W, Ebert PR (2020) Suppression of a core metabolic enzyme dihydrolipoamide dehydrogenase (dld) protects against amyloid beta toxicity in C. elegans model of Alzheimer’s disease. Genes Dis. 10.1016/j.gendis.2020.08.004 10.1016/j.gendis.2020.08.004 PMC8427249 34522713

[83] Naghizadeh S, Mansoori B, Mohammadi A, Sakhinia E, Baradaran B (2019). Gene silencing strategies in cancer therapy: an update for drug resistance. Curr Med Chem.

[84] Uludag H, Parent K, Aliabadi HM, Haddadi A (2020). Prospects for RNAi therapy of COVID-19. Front Bioeng Biotechnol.

